# Efficacy and safety of ketamine in total hip arthroplasty: a systematic review

**DOI:** 10.1186/s13018-026-06972-4

**Published:** 2026-06-01

**Authors:** Taotao Xu, Weiji Yang, Lei Chen, Jiayao Zhu, Xiheng Lu, Yiqing Ling, Ju Li, Xiangjiao Yi, Zhenyu Shi, Bo Zhu

**Affiliations:** 1 The First Affiliated Hospital of Zhejiang Chinese Medical University (Zhejiang Provincial Hospital of Chinese Medicine), 54 Youdian Road, Hangzhou, 310006 Zhejiang Province China; 2 Zhejiang Chinese Medical University, 548 Binwen Road, Hangzhou, 310053 Zhejiang Province China

**Keywords:** Ketamine, Esketamine, Total hip arthroplasty, Postoperative delirium

## Abstract

**Background:**

Total hip arthroplasty (THA) is a common procedure for end-stage hip disorders, yet optimal perioperative pain management remains challenging. Ketamine and its S-enantiomer, esketamine, have been proposed as adjuncts in multimodal analgesia due to their opioid-sparing effects and unique mechanisms. However, existing evidence on their efficacy and safety in THA patients is conflicting and has not been systematically reviewed.

**Methods:**

A systematic search of PubMed, Cochrane Library, EMBASE, and Web of Science was conducted from inception to February 2026. Studies were included if they involved THA patients receiving perioperative ketamine or esketamine and reported outcomes related to pain, opioid use, delirium, functional recovery, or adverse events. Quality was assessed using the Cochrane Risk of Bias 2 (RoB 2) tool for randomized controlled trials and the Newcastle–Ottawa Scale (NOS) for cohort studies.

**Results:**

Thirteen studies (10 RCTs, 3 cohort studies) encompassing 693,978 patients were included. Although several studies reported reductions in postoperative pain scores and opioid consumption, as well as improvements in early functional recovery and emotional status, these findings were not universal, and heterogeneity across study protocols precludes definitive conclusions. Findings on postoperative delirium were conflicting: one large RCT reported a significant reduction, while a retrospective study suggested increased risk with ketamine use. No serious adverse events were reported; however, psychotomimetic effects such as hallucinations and nightmares were more common in some ketamine groups compared to controls.

**Conclusions:**

Perioperative ketamine may offer benefits in analgesia, opioid sparing, and functional recovery after THA, but its effects on delirium are inconsistent and may depend on dosing and patient characteristics. Individualized administration and cautious use in high-risk elderly patients may be considered, though further research is needed to clarify optimal protocols.

**Supplementary Information:**

The online version contains supplementary material available at 10.1186/s13018-026-06972-4.

## Background

Total hip arthroplasty is one of the most effective surgical procedures for treating end-stage hip osteoarthritis, femoral head necrosis, rheumatoid arthritis, and femoral neck fractures, significantly alleviating pain, correcting joint deformities, restoring joint function, and improving patients’ quality of life [1]. With the global aging population and the increasing prevalence of hip disorders, the demand for THA continues to rise annually. Although surgical techniques, anesthesia management, and perioperative rehabilitation strategies have been continuously optimized in recent years, postoperative complications remain critical factors affecting early recovery and long-term outcomes [2].

Ketamine, a non-competitive N-methyl-D-aspartate (NMDA) receptor antagonist, has been widely used for clinical anesthesia induction and maintenance since the 1960 s [3]. Its unique pharmacological profile enables it to effectively inhibit central sensitization, modulate glutamatergic neurotransmission, reduce inflammatory responses, and potentially exert neuroprotective effects at sub-anesthetic doses (typically defined as intravenous bolus of 0.1–0.5 mg/kg or infusion of 0.5–2 µg kg⁻¹ min⁻¹, well below the induction dose of 1–2 mg/kg) [4]. In recent years, the S-enantiomer of ketamine—S-ketamine (hereafter referred to as esketamine)—has gained increasing attention due to its higher analgesic potency (approximately twice that of ketamine), shorter half-life, and fewer psychotomimetic side effects. It has been extensively applied in perioperative analgesia, treatment-resistant depression, and other fields [5].

In the perioperative management of THA, pain control is a critical determinant of early mobilization, functional recovery, and hospital length of stay. Opioids remain the mainstay of postoperative analgesia; however, their dose-related adverse effects—including nausea and vomiting, respiratory depression, ileus, urinary retention, and opioid-induced hyperalgesia—significantly limit their clinical utility [6]. Furthermore, opioid use may increase the risk of postoperative delirium, particularly in elderly patients [6]. Therefore, identifying adjunctive agents that can reduce opioid consumption while optimizing analgesic quality has become a research priority in perioperative management.

Ketamine and its isomers, with their unique analgesic mechanisms and opioid-sparing effects, are considered promising components of multimodal analgesia. Existing evidence suggests that perioperative ketamine administration may reduce postoperative pain scores, decrease opioid consumption, shorten time to first ambulation, improve postoperative joint function recovery, and positively influence postoperative delirium and emotional status [7]. However, findings across studies remain heterogeneous: some studies have demonstrated that ketamine significantly reduces the incidence of postoperative delirium, while others have found no significant benefit, and some retrospective cohort studies have even suggested an association between ketamine use and an increased risk of delirium [8]. Similarly, inconsistent results have been reported regarding analgesic efficacy, opioid-sparing effects, functional recovery, and adverse events. This heterogeneity may be attributable to differences in ketamine dosage, timing of administration, route of administration, patient population characteristics, and concomitant analgesic regimens.

To date, no systematic review has comprehensively integrated and evaluated the existing evidence on perioperative ketamine use in THA, particularly regarding its efficacy and safety across different administration protocols and patient subgroups.

Therefore, this systematic review aims to comprehensively evaluate the efficacy and safety of perioperative ketamine (including S-ketamine/esketamine) in patients undergoing THA, with a focus on postoperative pain control, opioid consumption, postoperative delirium, functional recovery, emotional status, and adverse events. The findings are intended to provide evidence-based guidance for optimizing perioperative analgesic strategies and to inform directions for future research.

## Methods

This review was reported in accordance with the Preferred Reporting Items for Systematic Reviews and Meta-Analyses (PRISMA) 2020 [9]. This study was registered in the International Prospective Register of Systematic Reviews (CRD420261355733).

### Search strategies

A systematic review according to the PRISMA guidelines was performed. A systematic search was performed in PubMed, Cochrane Library, EMBASE, and Web of Science from database inception to February 2026. To identify potentially relevant studies, a search strategy combining Medical Subject Headings terms and free-text words was employed, including: “total hip arthroplasty,” “hip replacement,” “ketamine,” “esketamine,” “S-ketamine,” “postoperative analgesia,” and “postoperative delirium.” The detailed search strategies for each database are provided in Supplementary Material 1. No language restrictions were applied, but grey literature (e.g., conference proceedings, trial registries) was not systematically searched, which is acknowledged as a limitation.

### Inclusion and exclusion criteria

The inclusion criteria for this systematic review were as follows: patients undergoing total hip arthroplasty; perioperative administration of ketamine, S-ketamine, or esketamine as the intervention; study designs including randomized controlled trials, cohort studies, or case-control studies; and reporting of at least one outcome related to postoperative pain, opioid consumption, postoperative delirium, functional recovery, or adverse events.

Studies were excluded if they were available only as abstracts without full text, if data could not be extracted or analyzed, if they included mixed surgical procedures (e.g., both hip and knee arthroplasty) without separately reporting data for THA patients, or if they were reviews, case reports, editorials, or conference abstracts.

### Data extraction

Two reviewers independently screened the titles and abstracts of all identified studies. Any disagreements were resolved through discussion with a third reviewer. The reference lists of the included studies were also manually reviewed to identify additional potentially eligible articles.

For each study meeting the inclusion criteria, the following information was extracted: first author, publication year, country, study design, sample size, patient age characteristics, ketamine (including S-ketamine/esketamine) regimen, control group intervention, and primary and secondary outcome measures. When a study included patients undergoing both THA and other surgical procedures (e.g., total knee arthroplasty), only data specific to the THA subgroup or separately reported data for THA patients were extracted and analyzed. Studies that did not provide separate outcome data for THA patients were excluded from this review.

### Quality assessment

The methodological quality of the included studies was assessed using the Cochrane Risk of Bias 2 (RoB 2) tool for randomized controlled trials (RCTs) and the Newcastle-Ottawa Scale (NOS) for cohort studies. The RoB 2 tool evaluates five domains: randomization, deviations from intended interventions, missing outcome data, measurement of the outcome, and selection of the reported result. The NOS assesses study quality across three categories: selection, comparability, and outcome. All assessments were performed independently by two reviewers. Disagreements during the evaluation process were resolved through discussion with a third author to ensure consistency and accuracy.

## Results

### Characteristics of included studies

**Fig. 1:**
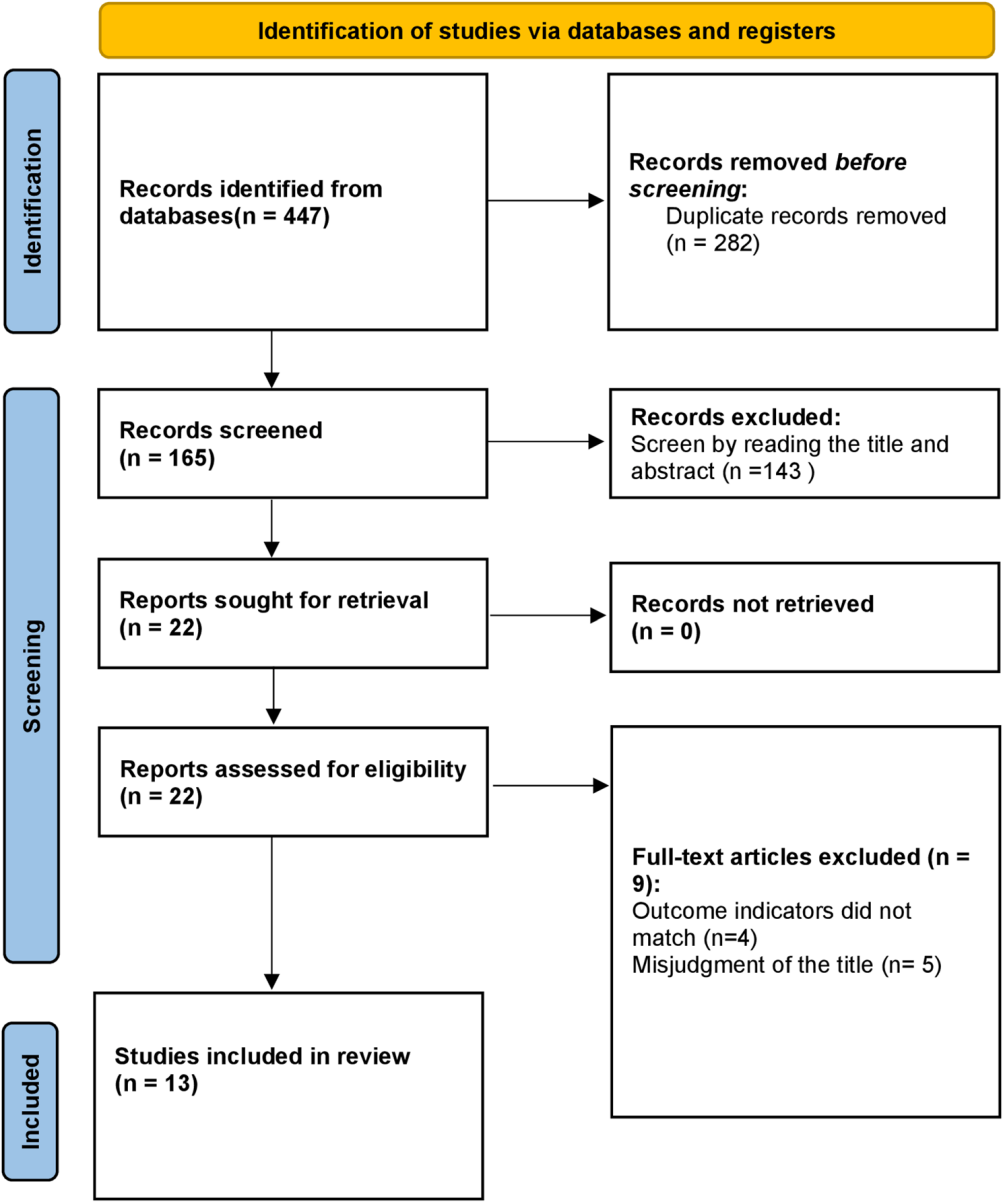
PRISMA flow chart

A total of 13 studies [10–22] were included in this systematic review, comprising 10 randomized controlled trials (RCTs) and 3 large retrospective cohort studies, published between 2009 and 2026. All studies evaluated the effects of perioperative ketamine (including its isomers S-ketamine/esketamine) on clinical outcomes in patients undergoing total hip arthroplasty (THA). The sample sizes of the included studies varied considerably, ranging from small RCTs with dozens of patients to large database analyses with over 500,000 patients. Notably, the studies by Cozowicz et al. [10] and Memtsoudis et al. [12], based on the US Premier Perspective database, included 181,182 patients with obstructive sleep apnea and 512,393 THA patients, respectively, providing large-scale real-world evidence on ketamine use. The remaining RCTs were primarily conducted in China, France, and Denmark, and mostly focused on elderly patient populations. Ketamine administration routes included single intraoperative bolus, intraoperative continuous infusion, and postoperative patient-controlled analgesia (PCA). Details can be seen in Table 1. The screening possession can be seen in Fig. 1.

**Table 1:** Characteristics of included studies

Study	Country	Study design	Sample size (T/C)	Patient age (years)	Ketamine regimen	Control group	Outcome measures (consolidated)
Cozowicz et al. [10]	USA	Retrospective cohort study	Total sample: 181,182 (ketamine users: *n* = 10,349)	Median age: 64–65 years (IQR 58–72)	Ketamine as part of multimodal analgesia for postoperative pain, dose not specified	Opioid-only patients	Opioid consumption; Opioid-related adverse events; Respiratory complications; Resource utilization (LOS, costs)
Dai et al. [21]	China	Randomized controlled trial	120 cases (40/40/40)	72.5 ± 6.1 (60–89 years)	Sub-anesthetic dose of esketamine (0.2 mg/kg IV) combined with PENG block	Group B: Sub-anesthetic dose esketamine combined with lumbar plexus block; Group C: Sub-anesthetic dose esketamine with general anesthesia	Anesthesia recovery quality (onset/recovery/extubation time); Hemodynamics (MAP/HR); Pain scores; Sedation scores; Postoperative delirium; Adverse events
Ma et al. [19]	China	Randomized controlled trial	130/130	Mean 69 years	Induction: 0.20 mg/kg; Intraoperative infusion: 0.125 mg/kg/h; Postoperative analgesia pump: 0.5 mg/kg (48 h)	Normal saline	Postoperative delirium (incidence, subtype)
Martinez et al. [13]	France	Randomized, double-blind, controlled study	142 cases (groups: placebo 38, ketamine 34, pregabalin 35, combination 35)	Mean age 59–64 years (slight variation between groups)	IV 0.5 mg/kg at induction, followed by intraoperative continuous infusion 3 µg/kg/h until end of surgery	Placebo (oral lactose capsules + IV saline)	Opioid consumption; Pain scores; Hyperalgesia; Opioid-related side effects; Ketamine-related side effects
Memtsoudis et al. [12]	USA	Retrospective cross-sectional cohort study	THA: 512,393;	Median age: THA approx. 64–67 years	Ketamine as one modality in multimodal analgesia, dose/route/timing not specified	Opioid-only patients	Opioid consumption; Hospital LOS; Hospital costs; Opioid-related adverse events
Min et al. [22]	China	Randomized controlled trial	67/65	≥ 60 years, mean 73.9 years	Esketamine 2.5 mg/kg in 100 ml saline for PCIA (background 2 ml/h, PCA 3 ml, lockout 15 min)	Sufentanil 2.5 µg/kg in 100 ml saline for PCIA (same parameters)	Pain scores; Functional recovery (time to first ambulation, ambulation distance); Analgesic requirements (PCA attempts); Hospital LOS; Adverse events; Inflammatory markers (IL-6, CRP); Psychological status (HAD); Hip function (Harris score)
Qu et al. [18]	China	Randomized controlled trial	135 cases (45 per group)	18–85 years	Postoperative PCIA: Esketamine 1.5 mg/kg + Flurbiprofen axetil 250 mg, diluted to 150 ml, background 3 ml/h, bolus 2 ml, lockout 15 min	Sufentanil group (2.0 µg/kg + Flurbiprofen axetil 250 mg) and FICB group (0.2% ropivacaine)	Pain scores; Psychological status (SAS/SDS); Satisfaction; Hip function; Functional recovery (ambulation time, LOS); Analgesic requirements; Motor/sensory block; Adverse events
Rasmussen et al. [15]	Denmark	Randomized, double-blind, placebo-controlled	24/18	Median age: 72 years (combination group), 70 years (control group)	Preoperative IV ketamine 0.15 mg/kg (S-ketamine), combined with gabapentin, dexamethasone, acetaminophen, and ketorolac	Acetaminophen + ketorolac + placebo	Opioid consumption; Pain intensity; PONV; Sedation; Dizziness; Hallucinations; Antiemetic (ondansetron) requirements
Remerand et al. [14]	France	Prospective, randomized, controlled, double-blind study	79/75	64 ± 13 (T)/65 ± 14 (C)	IV 0.5 mg/kg before incision, followed by continuous infusion 2 µg kg⁻¹ min⁻¹ for 24 h postop	Saline bolus + infusion	Opioid consumption; Pain scores; Chronic pain incidence; Functional recovery (ambulation, assistive devices); PONV; Psychomimetic side effects; Hospital LOS
Wei et al. [20]	China	Randomized double-blind study	38/34	Median 54 (46, 63)/51 (39, 61) years	Esketamine 100 mg + Dexmedetomidine 100 µg + Tropisetron 10 mg, diluted to 150 ml, PCA settings: background 3 ml/h, bolus 3 ml, lockout 30 min	Fentanyl 0.7 mg + Dexmedetomidine 100 µg + Tropisetron 10 mg, diluted to 150 ml, same PCA settings	Pain scores; Analgesic requirements; Sleep quality (PSQI); Adverse events; Postoperative LOS
Weinstein et al. [11]	USA	Retrospective cohort study	41,766 (total sample, delirium group 922 cases)	Median [IQR]: No delirium 66 [58–73], Delirium 78 [72–83]	Intraoperative ketamine (single dose), postoperative ketamine infusion	Patients not receiving ketamine	Postoperative delirium; Hospital LOS; Association of medications with delirium; Association of anesthesia type with delirium; Association of comorbidities with delirium
Zhao et al. [17]	China	Prospective, randomized, double-blind, controlled trial	57/55	65–80 years	Esketamine 0.72 mg/kg added to PCIA (combined with sufentanil), background infusion 15 µg/kg/h for 48 h	Sufentanil group (sufentanil only 2 µg/kg PCIA)	Sleep quality (sleep disturbance incidence, PSQI); Psychological status (anxiety, depression); Cognitive function; Analgesic requirements; Postoperative delirium; Postoperative recovery quality (QoR-15); Adverse events
Zhu et al. [19]	China	Single-center, prospective, randomized, double-blind, placebo-controlled trial	186/186	≥ 60 years (median approx. 67–68 years)	Intraoperative IV S-ketamine 0.2 mg kg⁻¹ h⁻¹ for 1 h; Postoperative PCIA with S-ketamine 1.0 mg kg⁻¹ for 48 h	Normal saline (intraoperative infusion + PCIA without S-ketamine)	Postoperative delirium (incidence, episodes, onset time, duration, severity, subtype); Pain scores; Opioid consumption; Analgesic requirements; Sleep quality; Functional outcomes; Hospital LOS; Adverse events

Given the anticipated substantial clinical heterogeneity across studies in terms of ketamine dosing regimens, administration protocols, patient populations, and outcome measurement tools, a quantitative meta-analysis was not planned. Instead, findings are presented as a structured narrative synthesis, with results grouped by outcome domain and study design. This approach allows for a more nuanced interpretation of the evidence while avoiding inappropriate pooling of heterogeneous data.

### Postoperative analgesic effect

While there was some heterogeneity in pain assessment across the included studies, findings on postoperative analgesia can be grouped according to whether ketamine demonstrated a positive effect.

Studies reporting positive analgesic effects. Four RCTs reported that ketamine significantly improved postoperative pain outcomes. Rasmussen et al. [15] found that a multimodal analgesic regimen including ketamine significantly reduced overall postoperative pain at rest (*P* = 0.042) and during movement (*P* = 0.027), and nearly eliminated moderate to severe pain. Studies by Ma et al. [19]and Zhu et al. [16] both indicated that patients receiving esketamine had significantly lower movement-evoked pain scores on postoperative days 1 and 2 compared to controls (*P* < 0.05 and *P* < 0.001, respectively). Dai et al. [21] (using a subanesthetic dose of esketamine 0.3 mg/kg intraoperatively) observed that esketamine combined with pericapsular nerve group (PENG) block resulted in significantly lower VAS scores at 2, 6, and 12 h postoperatively compared to controls (*P* < 0.001). Additionally, although Remerand et al. [14] did not find that ketamine significantly improved acute pain scores, they reported that it significantly reduced the area of secondary hyperalgesia around the surgical incision (318 ± 59 mm vs. 451 ± 59 mm, *P* = 0.02), suggesting that ketamine may exert analgesic effects by inhibiting central sensitization.

Studies reporting no significant differences. In contrast, several studies found no significant improvement in postoperative pain scores with ketamine. Martinez et al. [13] and Min et al. [22] reported no significant differences in pain scores at rest or during movement between the ketamine/esketamine groups and control groups. Wei et al. [20] also found no difference in early postoperative resting pain, although they did observe significantly lower resting pain in the esketamine group at 24–48 h postoperatively (*P* = 0.018).

### Opioid consumption

The impact of ketamine on opioid consumption varied across studies, potentially related to differences in administration timing, concomitant analgesic regimens, and study population bias. Five RCTs suggested a significant opioid-sparing effect of ketamine. Studies by Remerand et al. [14] and Martinez et al. [13] both indicated that morphine consumption was significantly lower in the ketamine groups compared to placebo groups. In the study by Remerand et al. [14] (*n* = 154), 24-hour morphine consumption was 14 ± 13 mg in the ketamine group versus 19 ± 12 mg in the placebo group (*P* = 0.004); the difference at 48 h was not statistically significant (*P* = 0.121). Martinez et al. [13] reported that 48-hour morphine consumption was significantly lower in the ketamine group (*P* < 0.001). Ma et al. [19] found that intraoperative remifentanil consumption was significantly reduced in the esketamine group (1373.1 ± 220.5 µg vs. 1465.4 ± 215.8 µg, $\:P<0.05$). The study by Wei et al. [20] showed that cumulative morphine equivalent consumption over 48 h postoperatively was significantly lower in the esketamine group compared to the fentanyl group (5 mg vs. 10 mg, $\:P=0.01$). Zhao et al. [17]also reported that the proportion of patients requiring rescue analgesia within 48 h postoperatively was significantly lower in the esketamine group (12.3% vs. 31.0%, $\:P=0.016$). Additionally, the large retrospective study by Memtsoudis et al. [12]showed that patients receiving multimodal analgesia including ketamine (more than two modalities) had a 12.5% reduction in total opioid prescription (95% CI −13.5% to −11.5%). However, in the study by Rasmussen et al. [15], although 24-hour morphine consumption was lower in the multimodal group (13 mg vs. 22 mg), the difference was not statistically significant (*P* = 0.085). Notably, two retrospective cohort studies observed opposite trends. Cozowicz et al. [10]found that ketamine use was associated with a 3.8% increase in opioid consumption, and Memtsoudis et al. [12] also reported in multivariate regression analysis that ketamine use alone was positively correlated with opioid prescription (+ 4.4%). Both authors suggested this likely reflects indication bias, where ketamine is preferentially used in patients with poorly controlled pain and higher opioid requirements.

### Postoperative complications

The effect of ketamine on postoperative complications presents a complex picture, with postoperative delirium (POD) being the most prominent and controversial outcome.

Evidence from RCTs on POD: The large RCT by Zhu et al. [16] provided strong evidence that S-ketamine significantly reduced the incidence of POD within the first 3 postoperative days in elderly patients (8.06% vs. 20.43%, adjusted OR = 0.29, 95% CI 0.14–0.63, *P* = 0.002, number needed to treat = 8). However, two other RCTs by Ma et al. [19]and Zhao et al. [17] did not find that esketamine significantly reduced POD incidence.

Evidence from retrospective cohort studies on POD: In contrast, the retrospective cohort study by Weinstein et al. [11] (*n* = 1,738) found that both intraoperative (OR = 1.27, 95% CI 1.01–1.59, *P* = 0.040) and postoperative (OR = 10.59, 95% CI 5.26–19.91, *P* < 0.001) ketamine use were significantly associated with an increased risk of POD, with a trend suggesting a dose-response relationship. It should be noted, however, that retrospective cohort studies are susceptible to indication bias and unmeasured confounding; therefore, this association does not establish a causal relationship and carries lower evidentiary weight than RCT findings.

Evidence on other complications (from retrospective studies): Regarding other complications, two large retrospective studies consistently showed that multimodal analgesia including ketamine was associated with reduced postoperative complication risks. Cozowicz et al. [10] reported that multimodal analgesia was associated with significant reductions in the need for mechanical ventilation (OR = 0.23, 95% CI 0.16–0.32), ICU admission (OR = 0.60, 95% CI 0.48–0.75), and gastrointestinal complications (OR = 0.65, 95% CI 0.53–0.78). Memtsoudis et al. [12] also found that patients receiving ketamine combined with multiple analgesic modalities had significantly lower rates of respiratory complications (OR = 0.81, 95% CI 0.70–0.94), gastrointestinal complications (OR = 0.74, 95% CI 0.65–0.84), and urological complications (OR = 0.79, 95% CI 0.70–0.89).

### Functional recovery and resource utilization

Regarding functional recovery, multiple studies indicated that ketamine may facilitate early postoperative functional recovery. Remerand et al. [14] found that the proportion of patients still requiring walking aids at 30 days postoperatively was significantly lower in the ketamine group compared to the placebo group (31% [22/71] vs. 56% [37/66], *P* = 0.0035), representing a 45% relative risk reduction. Min et al. [22] and Qu et al. [18] also reported that the time to first ambulation was significantly earlier in the esketamine groups compared to control groups ($\:P<0.05$ and $\:P<0.001$, respectively). Studies by Zhu et al. [16] and Min et al. [22] both showed that early postoperative (day 3 to 1 month) activities of daily living (Barthel Index) and hip function scores (Harris Hip Score) were significantly better in the ketamine groups than in control groups ($\:P<0.001$ and $\:P<0.05$, respectively). Zhao et al. [17] also found that quality of recovery scores (QoR-15) on postoperative days 1–3 were significantly higher in the esketamine group ($\:P<0.05$). Regarding hospital length of stay(LOS) and costs, large retrospective studies consistently showed that multimodal analgesic strategies including ketamine were associated with reduced hospital stay. Cozowicz et al. [10] reported an 11.8% reduction in length of stay (95% CI −13.0% to −10.7%), and Memtsoudis et al. [12] (2018) reported a 12.1% reduction (95% CI −12.8% to −11.5%), with median stay decreasing from 3 to 2 days. However, most RCTs, such as those by Remerand et al. [14], Wei et al. [20], and Zhu et al. [16], did not find that ketamine significantly shortened hospital stay. Regarding hospital costs, Cozowicz et al. [10] and Memtsoudis et al. [12] reported small but significant reductions (3.2% and 2.1%, respectively).

Notably, a discrepancy emerged between study designs regarding hospital length of stay. While two large retrospective studies reported significant reductions in hospital stay associated with multimodal analgesia including ketamine (11.8% and 12.1% reductions, respectively) [10, 12], most RCTs did not identify a significant difference [14, 16, 20]. Several factors may explain this inconsistency. First, large retrospective studies, with sample sizes exceeding 500,000 patients, have high statistical power to detect small differences in length of stay, whereas individual RCTs were typically underpowered for this outcome. Second, retrospective database studies often capture standardized discharge protocols within large healthcare systems, potentially reflecting system-level efficiencies rather than the direct effect of ketamine. Conversely, RCTs frequently employ stricter discharge criteria and may have shorter baseline lengths of stay, leaving less room for improvement. Third, the multimodal analgesic protocols in retrospective studies included multiple non-opioid modalities, making it difficult to isolate the specific contribution of ketamine to the observed reductions.

### Emotional state and sleep quality

Regarding emotional state, three studies evaluated the impact of ketamine on mood, all with positive results. Studies by Min et al. [22], Qu et al. [18], and Zhao et al. [17]all showed that anxiety (HAMA/SAS) and depression (HAMD/SDS) scores in the early postoperative period (3 days to 1 week) were significantly lower in the esketamine groups compared to control groups ($\:P<0.05$). Qu et al. [18]also found that patient satisfaction was significantly higher in the esketamine group ($\:P=0.014$). Regarding sleep quality, results were mixed. The study by Zhao et al. [17] showed that esketamine significantly reduced the incidence of sleep disturbance on postoperative days 1–3 (day 1: 28.1% vs. 56.4%, $\:P=0.002$) and improved subjective sleep quality scores. However, studies by Ma et al. [19] and Wei et al. [20] did not find that esketamine significantly improved postoperative sleep quality.

### Adverse events and safety

Overall, at the doses used in clinical studies, ketamine demonstrated a favorable safety profile, with serious adverse events being rare. Regarding psychotomimetic adverse effects, most RCTs, such as those by Martinez et al. [13], Remerand et al. [14], and Qu et al. [18], reported low incidences of hallucinations, nightmares, and other psychiatric symptoms in the ketamine groups, with no significant differences compared to control groups. However, in the large RCT by Zhu et al. [16], although the proportions of patients experiencing nightmares (3.8%), dizziness (5.9%), and hallucinations (5.4%) in the S-ketamine group were low, they were significantly higher than in the placebo group ($\:P<0.05$). Regarding postoperative nausea and vomiting (PONV), studies by Min et al. [22] and Qu et al. [18] showed that the incidence of PONV was significantly lower in the esketamine groups compared to opioid control groups (15% vs. 40%, $\:P<0.001$; $\:P<0.05$, respectively). Dai et al. [21] also reported that esketamine combined with nerve block reduced the risk of PONV. Regarding dizziness, in the study by Ma et al. [19], the incidence of transient postoperative dizziness was higher in the esketamine group (39.2% vs. 22.3%, $\:P<0.05$), while Wei et al. [20] found that the incidence of urinary retention was significantly lower in the esketamine group (0% vs. 12.5%, $\:P=0.03$). No serious adverse events related to ketamine, such as respiratory depression, cardiovascular accidents, or intraoperative awareness, were reported in any of the included studies.

### Quality assessment

The methodological quality of the included studies was assessed using the RoB 2 tool for the 10 randomized controlled trials and NOS for the 3 retrospective cohort studies.

Among the three included retrospective cohort studies, the overall methodological quality was high. The studies by Cozowicz et al. [10] and Memtsoudis et al. [12] achieved the maximum NOS score (9/9 stars). Both utilized the nationally representative Premier Healthcare database, ensuring reliable ascertainment of exposure and outcomes through standardized billing and diagnostic codes. They employed multilevel multivariable regression models to adequately control for confounders, including patient demographics, comorbidities, and hospital-level factors. Furthermore, due to their reliance on complete inpatient billing data, follow-up was comprehensive with no loss to follow-up. The study by Weinstein et al. [11] scored 8 out of 9 stars, also classifying it as high quality. This study leveraged detailed electronic medical records from a single high-volume institution, providing reliable exposure and outcome data. It employed comprehensive multivariable regression models and multiple imputation to address confounding and missing data. The only point deducted was for the adequacy of follow-up (Outcome domain), as the study did not explicitly report using a standardized screening tool for delirium for all patients, which may introduce potential bias in outcome ascertainment.

Regarding the 10 included randomized controlled trials, the overall quality was also high. Four studies (Remerand et al. [14]; Zhu et al., [16]; Zhao et al. [17]; Ma et al., [19]) were rated as high quality, meeting all criteria across the five domains of the RoB 2 tool. Two studies (Martinez et al. [13]; Rasmussen et al. [15]), also considered high quality, primarily due to missing outcome data or incomplete reporting. Two studies (Qu et al., [18]; Wei et al. [20]) were rated as moderate quality, with the main limitation being the lack of blinding, which may introduce performance and detection bias. The remaining two studies (Dai et al. [21]; Min et al. [22]) were rated as low quality, exhibiting significant issues related to blinding, data completeness, and reporting, thus warranting cautious interpretation of their conclusions.

In summary, the majority of the included studies demonstrated high methodological rigor, providing reliable evidence on the efficacy and safety of perioperative analgesic management strategies. The details can be seen in supplement material 1 and 2.

## Discussion

This systematic review included 13 studies evaluating the efficacy and safety of perioperative ketamine (including esketamine) in patients undergoing total hip arthroplasty. The available evidence suggests that ketamine demonstrates positive value in postoperative analgesia, opioid sparing, functional recovery, and emotional improvement, while its effects on postoperative delirium and sleep quality remain controversial. These findings indicate that the clinical application of ketamine requires balancing benefits against risks, with full consideration of patient characteristics and differences in administration protocols.

Multiple RCTs suggested that ketamine reduces postoperative pain scores, particularly movement-evoked pain, while decreasing opioid consumption. This effect is rooted in ketamine’s role as a non-competitive NMDA receptor antagonist, which blocks central sensitization—a key mechanism underlying postoperative hyperalgesia and allodynia.

 [23, 24]. Better pain control reduces opioid demand, and decreased opioid consumption further mitigates opioid-related adverse effects such as nausea, sedation, and ileus, creating a virtuous cycle that promotes early mobilization and functional recovery [25]. Notably, two large retrospective studies paradoxically associated ketamine use with increased opioid consumption [10, 12]. However, this likely reflects indication bias—ketamine being preferentially administered to patients with poorly controlled pain and inherently higher opioid requirements. This discrepancy underscores the irreplaceable role of RCT evidence in causal inference and highlights the importance of distinguishing efficacy (demonstrated in RCTs) from real-world practice patterns (captured in observational studies) [7, 26, 27].

The effect of ketamine on postoperative delirium represents the most concerning and controversial finding in this review, primarily due to a direct conflict between the available RCT and retrospective cohort data. From a methodological perspective, these study designs carry different evidentiary weight for causal inference. The large RCT by Zhu et al. [16] provided high-quality evidence that S-ketamine significantly reduced delirium incidence in elderly THA patients, consistent with its anti-inflammatory and neuroprotective properties [28–30]. Meanwhile, excitotoxicity resulting from overexcitation of the glutamatergic system also plays a role in delirium pathogenesis, and ketamine as an NMDA receptor antagonist can directly intervene at this juncture [31]. In contrast, the retrospective cohort study by Weinstein et al. [11]reported that ketamine use was associated with an increased risk of delirium, with a dose-response relationship. However, due to the inherent limitations of observational designs—including indication bias (ketamine preferentially given to higher-risk patients), unmeasured confounding, and potential misclassification of delirium—this finding should be interpreted as hypothesis-generating rather than evidence of a causal harmful effect. The divergence between the RCT and retrospective findings likely stems from ketamine’s bidirectional effects on the central nervous system, which may be dose- and context-dependent. At sub-anesthetic doses, it exerts neuroprotection by inhibiting excessive NMDA receptor activation; however, at higher doses or in susceptible individuals, it may induce psychotomimetic symptoms by disrupting GABAergic pathways and overexciting glutamatergic transmission [32, 33]. Administration route is critically important: single intraoperative bolus versus postoperative continuous infusion have different pharmacokinetic profiles, with the latter potentially leading to higher cumulative exposure—consistent with the elevated risk observed in the postoperative infusion group by Weinstein et al. [11]. Therefore, while a single high-quality RCT supports a protective effect of a single intraoperative bolus of S-ketamine on POD, this evidence has not yet been replicated, and the conflicting signal from observational data precludes definitive conclusions. Clinical practice should not simplistically categorize ketamine as either deliriogenic or delirium-preventing, but rather individualize decisions based on patient characteristics and administration protocols, with cautious use in high-risk elderly patients receiving prolonged infusions [34, 35].

The safety profile of ketamine was generally favorable, with no serious adverse events reported across included studies. Some studies suggested an association with reduced postoperative nausea and vomiting [18, 21, 22] consistent with prior meta-analyses [36, 37]; second, ketamine itself may possess direct antiemetic properties, potentially involving modulation of central neurotransmitter pathways, though the precise mechanisms remain to be fully elucidated. On the other hand, ketamine-specific psychotomimetic side effects (hallucinations, nightmares) and dizziness warrant attention—these effects are inseparable from its central mechanism of action: NMDA receptors are widely distributed in the cerebral cortex, hippocampus, and prefrontal cortex, participating in the regulation of cognition, emotion, and perception [38, 39]; ketamine’s antagonism of NMDA receptors can disrupt normal function in these brain regions, inducing dissociative symptoms. The occurrence of dizziness may be related to antagonism of NMDA receptors in the vestibular system, interfering with the transmission and integration of vestibular signals to the central nervous system [40]. However, this outcome was not the primary focus of most studies, and findings should be interpreted with caution. Psychotomimetic effects (hallucinations, nightmares) and dizziness warrant attention. In the large RCT by Zhu et al. [16], although the absolute incidence of psychotomimetic side effects in the S-ketamine group was not high, it was significantly higher than in the placebo group, suggesting that even at conventional doses, this issue cannot be completely overlooked. It is worth emphasizing that none of the included studies reported serious adverse events related to ketamine, providing fundamental safety assurance for its perioperative application in THA.

Significant heterogeneity exists among the studies included in this review, and this observed heterogeneity offers important clinical insights. Ketamine administration protocols, choice of control interventions, patient population characteristics, and outcome measurement tools may all influence study conclusions. This heterogeneity suggests that there is no one-size-fits-all approach to ketamine application in THA; rather, refinement should be based on understanding its pharmacological mechanisms in conjunction with specific clinical contexts.

Given the substantial heterogeneity and conflicting evidence across included studies, definitive clinical recommendations cannot be made. Future research should prioritize well-designed dose-response studies to define optimal ketamine regimens, including comparisons between single intraoperative bolus versus continuous infusion, timing of administration, and cumulative dose thresholds that balance analgesic efficacy against psychotomimetic risks. Patient selection criteria—particularly age, baseline cognitive function, preoperative opioid use, and inflammatory status—require further validation to identify subgroups most likely to benefit. The risk-benefit balance for elderly high-risk patients (age > 75 years, pre-existing cognitive impairment, multiple comorbidities) remains uncertain, and any potential use would require individual assessment with enhanced delirium monitoring. Direct head-to-head trials comparing ketamine and esketamine are needed, and future studies should stratify by anesthesia type (neuraxial vs. general) to examine potential effect modification. Finally, standardization of outcome measures is essential; unified diagnostic criteria for postoperative delirium (e.g., DSM-5 or CAM) and consistent assessment time points should be adopted to enable future quantitative synthesis and cross-study comparability.

Previous meta-analyses in mixed orthopedic populations (including both hip and knee arthroplasty) have reported overall beneficial effects of ketamine on postoperative pain and opioid consumption [34]. However, these syntheses pooled data across different procedures despite potential differences in surgical trauma and rehabilitation protocols between hip and knee arthroplasty. The present review focused exclusively on THA and did not perform a quantitative meta-analysis due to substantial clinical heterogeneity across included studies in ketamine dosing, administration timing, routes of delivery, and outcome measures. Our narrative synthesis reveals that beneficial effects are not uniformly consistent across THA-specific studies, and conflicting evidence on postoperative delirium—not consistently addressed in previous meta-analyses due to limited primary data at the time—remains unresolved. This review thus provides a procedure-specific synthesis highlighting heterogeneity as a central finding and identifies critical knowledge gaps for future research.

### Limitations

Several limitations of this systematic review should be acknowledged. First, substantial clinical heterogeneity across the included studies—stemming from differences in ketamine dosage (ranging from sub-anesthetic boluses to continuous infusions), timing of administration (intraoperative only versus combined intraoperative and postoperative), routes of delivery (single bolus, continuous infusion, or patient-controlled analgesia), patient populations (varying age ranges, comorbidity profiles, and baseline delirium risk), and outcome measurement tools (different pain scales, delirium screening instruments, and functional assessment metrics)—precluded a quantitative meta-analysis. Consequently, the findings are based on descriptive synthesis, which limits the ability to derive precise effect estimates or assess the robustness of pooled results.

Second, geographic and language bias may affect the generalizability of the findings. The majority of included studies were conducted in China, France, and Denmark, with only one study from the United States. Additionally, only English-language publications were retrieved, which may have introduced selection bias and limited the comprehensiveness of the evidence synthesis.

Third, the absence of a formal meta-analysis precluded assessment of publication bias. As with any systematic review, the possibility of publication bias remains a concern, given that studies with positive or statistically significant results are more likely to be published than those with null or negative findings.

Fourth, several outcomes of interest were reported in only a small number of studies. For instance, sleep quality was evaluated in only three studies, and emotional status in three studies, limiting the ability to draw robust conclusions. Furthermore, subgroup analyses based on delirium subtypes (hyperactive, hypoactive, mixed) or ketamine isomers (ketamine versus esketamine) could not be performed due to insufficient data.

Fifth, the small number of studies for certain outcomes and the lack of subgroup analyses limited the depth of synthesis. These limitations underscore that the conclusions of this review should be interpreted with caution and highlight the need for future studies with standardized protocols, unified outcome measures, and larger sample sizes to enable quantitative synthesis.

## Conclusion

This review mapped the available evidence on perioperative ketamine and esketamine use in total hip arthroplasty, identifying substantial heterogeneity in study protocols and conflicting findings across key outcomes. Regarding postoperative analgesia, opioid reduction, functional recovery, and emotional status, benefits were suggested by multiple RCTs, although these findings were not universally consistent.

In contrast, the evidence for postoperative delirium is limited and conflicting. A single high-quality RCT reported a protective effect of a single intraoperative bolus of S-ketamine, while one retrospective cohort study suggested an association with increased risk, particularly with postoperative continuous infusion. Due to the methodological limitations of observational data and the lack of replication of the RCT finding, the net effect of ketamine on delirium remains uncertain.

The available evidence is insufficient to provide definitive clinical recommendations. Instead, this review highlights critical knowledge gaps that warrant further investigation. Future research should focus on: (1) establishing optimal dosing protocols, including comparisons between single intraoperative bolus and continuous infusion; (2) validating patient selection criteria, particularly regarding age, baseline cognitive function, and delirium risk; (3) directly comparing ketamine isomers (ketamine versus esketamine); and (4) exploring whether the efficacy of ketamine differs between spinal and general anesthesia. Large-scale, well-designed RCTs with standardized outcome measures are urgently needed to address these gaps and inform evidence-based perioperative analgesic strategies.

## Supplementary Information

Below is the link to the electronic supplementary material.

Supplementary Material 1

Supplementary Material 2

## Data Availability

This study is a systematic review. All the data sourced from the articles listed in the tables within the manuscript.
